# Generative Pre-trained Transformer 4 makes cardiovascular magnetic resonance reports easy to understand

**DOI:** 10.1016/j.jocmr.2024.101035

**Published:** 2024-03-07

**Authors:** Babak Salam, Dmitrij Kravchenko, Sebastian Nowak, Alois M. Sprinkart, Leonie Weinhold, Anna Odenthal, Narine Mesropyan, Leon M. Bischoff, Ulrike Attenberger, Daniel L. Kuetting, Julian A. Luetkens, Alexander Isaak

**Affiliations:** a Department of Diagnostic and Interventional Radiology, University Hospital Bonn, Venusberg-Campus 1, 53127 Bonn, Germany; b Quantitative Imaging Lab Bonn (QILaB), University Hospital Bonn, Venusberg-Campus 1, 53127 Bonn, Germany; c University Hospital Bonn, Department of Medical Biometry, Informatics, and Epidemiology, Venusberg-Campus 1, 53127 Bonn, Germany

**Keywords:** Generative Pre-trained Transformers, Cardiovascular magnetic resonance, Artificial intelligence, Text simplification, Large language models

## Abstract

**Background:**

Patients are increasingly using Generative Pre-trained Transformer 4 (GPT-4) to better understand their own radiology findings.

**Purpose:**

To evaluate the performance of GPT-4 in transforming cardiovascular magnetic resonance (CMR) reports into text that is comprehensible to medical laypersons.

**Methods:**

ChatGPT with GPT-4 architecture was used to generate three different explained versions of 20 various CMR reports (n = 60) using the same prompt: “Explain the radiology report in a language understandable to a medical layperson”. Two cardiovascular radiologists evaluated understandability, factual correctness, completeness of relevant findings, and lack of potential harm, while 13 medical laypersons evaluated the understandability of the original and the GPT-4 reports on a Likert scale (1 “strongly disagree”, 5 “strongly agree”). Readability was measured using the Automated Readability Index (ARI). Linear mixed-effects models (values given as median [interquartile range]) and intraclass correlation coefficient (ICC) were used for statistical analysis.

**Results:**

GPT-4 reports were generated on average in 52 s ± 13. GPT-4 reports achieved a lower ARI score (10 [9–12] vs 5 [4–6]; p < 0.001) and were subjectively easier to understand for laypersons than original reports (1 [1] vs 4 [4,5]; p < 0.001). Eighteen out of 20 (90%) standard CMR reports and 2/60 (3%) GPT-generated reports had an ARI score corresponding to the 8th grade level or higher. Radiologists’ ratings of the GPT-4 reports reached high levels for correctness (5 [4, 5]), completeness (5 [5]), and lack of potential harm (5 [5]); with “strong agreement” for factual correctness in 94% (113/120) and completeness of relevant findings in 81% (97/120) of reports. Test-retest agreement for layperson understandability ratings between the three simplified reports generated from the same original report was substantial (ICC: 0.62; p < 0.001). Interrater agreement between radiologists was almost perfect for lack of potential harm (ICC: 0.93, p < 0.001) and moderate to substantial for completeness (ICC: 0.76, p < 0.001) and factual correctness (ICC: 0.55, p < 0.001).

**Conclusion:**

GPT-4 can reliably transform complex CMR reports into more understandable, layperson-friendly language while largely maintaining factual correctness and completeness, and can thus help convey patient-relevant radiology information in an easy-to-understand manner.

## Graphical abstract

**Figure:**
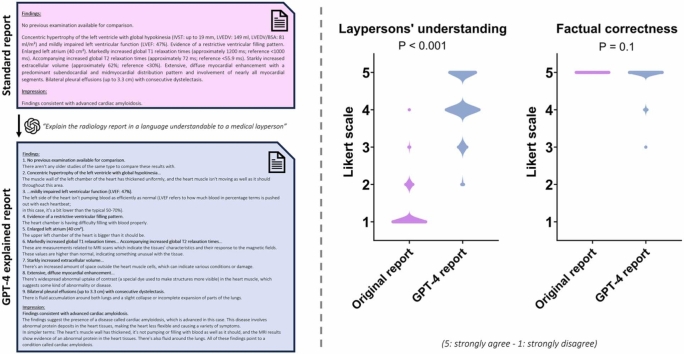

## 1 Introduction

Large language models (LLMs), such as Generative Pre-trained Transformer (GPT), have gained tremendous attention worldwide, reaching over 100 million users just 2 months after its launch [1]. Some potentially promising applications of GPT in radiology are in the areas of medical writing, clinical decision-making, education, and data analysis [2], [3], [4], [5], [6], [7], [8], [9]. OpenAI’s latest foundation model, GPT-4, has already demonstrated substantial advancements in terms of improved accuracy, reduced confabulation (creative gap-filling with false information), and a better understanding of detailed instructions compared to GPT-3.5 [10], [11], [12], [13].

A lack of understanding regarding basic radiology terminology is a common problem for many patients, which can foster misunderstanding and miscommunication. Typically, radiology findings are reported in a free-flow text format, employing technical and medical vocabulary that is necessary for precise communication between physicians. However, these reports are often cryptic and potentially misleading to individuals who lack medical background knowledge [14], [15], [16], [17]. One study demonstrated that only 4% of the radiology reports analyzed were understandable to the average adult in the United States [18]. These comprehensibility problems are even more pronounced in subspecialized imaging, which uses specific and often complex terminology that can be difficult to understand for both laypersons as well as physicians not specialized in that particular medical field. Cardiovascular magnetic resonance (CMR) reports can be challenging to understand due to their specific technical and anatomical language [19], [20].

General access to tools that allow the conversion of individual radiology reports into language that patients can understand is limited and usually requires additional resources. Thus, the widespread availability of LLMs could help patients more easily understand their health status, thereby promoting patient-centered health care [21], [22]. Although initial reports and pioneer studies have demonstrated promising results [23], [24], [25], [26], the applicability of LLMs in specialized and complex reports has been poorly investigated. The aim of this study was to evaluate GPT-4-explained CMR reports with particular emphasis on their factual correctness as well as their understandability to medical laypersons.

## 2 Materials and methods

**Fig. 1:**
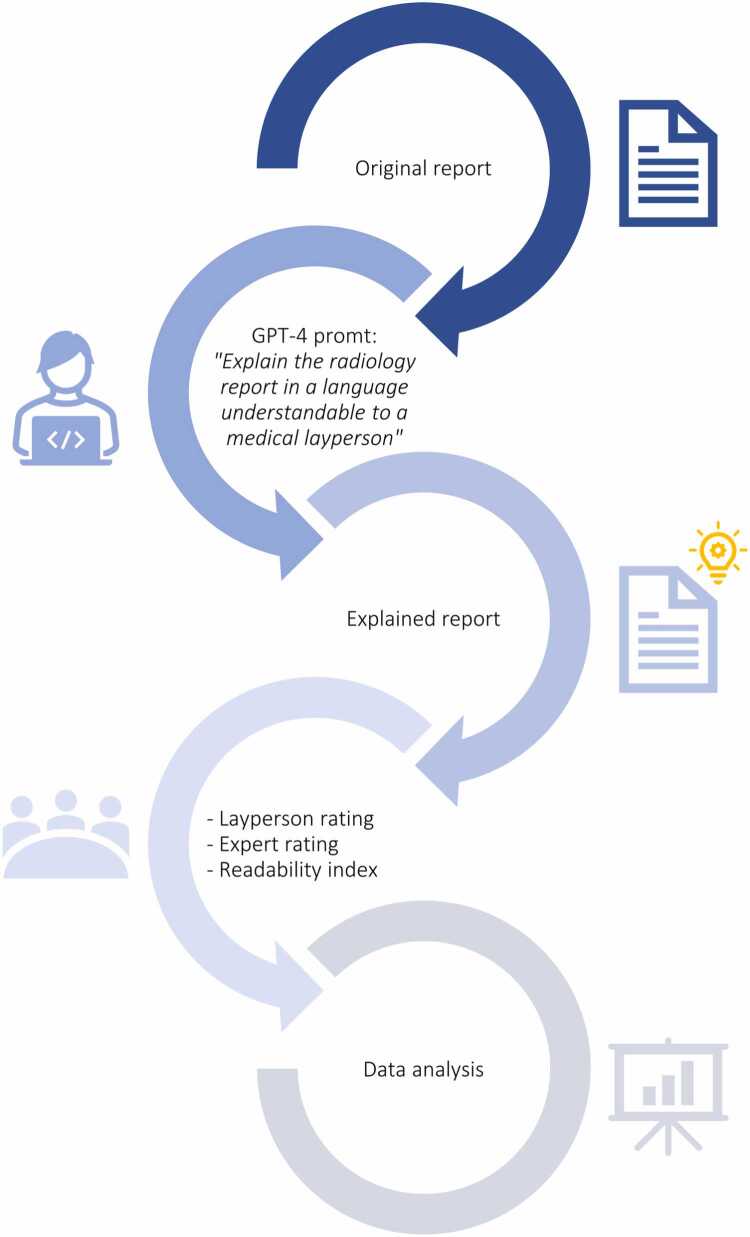
Visual summary of the study design. Based on original reports, 20 fictional cardiovascular magnetic resonance reports were created. Using GPT-4 (Generative Pre-trained Transformer 4), three simplified versions were generated for each original report using the same prompt. The generated versions were evaluated by medical laypersons (n = 13) and radiological experts (n = 2) using questionnaires. Quantitative readability analyses were conducted for original and simplified reports.

Approval of an institutional review board was not required because fictitious data were used. A visual summary of the study design is provided in Fig. 1.

### 2.1 Preparation of fictitious standard radiological reports

A board-certified native English-speaking radiologist with 9 years of experience in cardiovascular imaging (D.L.K.) created a set of 20 fictitious CMR reports in English. These reports were based on real reports and authentic clinical scenarios from routine clinical practice. No sensitive personal information from existing reports was used. The reports largely corresponded to typical CMR reports in terms of both structure and content. To ensure a diversity of reports in terms of form, content, and medical complexity, the reports were categorized thematically into four categories: nonischemic cardiomyopathy, ischemic cardiomyopathy, congenital heart disease, and structured reports. The reports in the first three categories were given in a free-text style, the reports in the last report category as a structured report. Five comprehensive reports were generated for each of the four report categories.

### 2.2 Generating explained reports using GPT-4

**Fig. 2:**
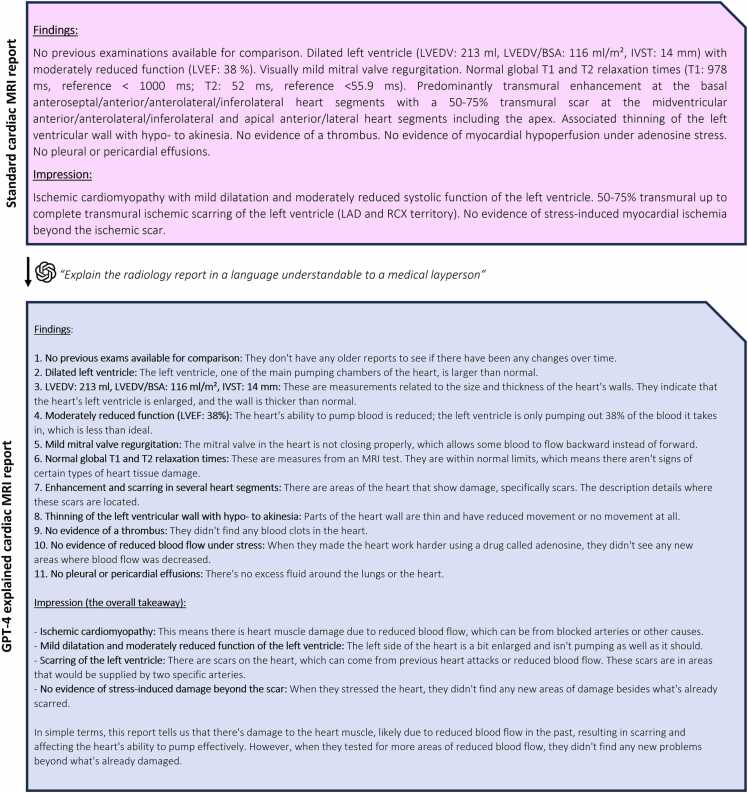
Example of a standard cardiovascular magnetic resonance report with its corresponding explained report generated by GPT-4 (Generative Pre-trained Transformer 4).BSA: body surface area, IVST: interventricular septal thickness, LAD: left anterior descending, LVEDV: left ventricular end-diastolic volume, LVEF: left ventricular ejection fraction, RCX: ramus circumflex artery.

ChatGPT based on GPT-4 architecture (version: August 3, 2023, OpenAI, San Francisco, California, ) was utilized to create a total of 60 simplified and explicated reports from the 20 standard CMR reports [11]. For this, a common prompt was defined that could be used by patients in real-life scenarios: “Explain the radiology report in a language understandable to a medical layperson” followed by the full text of the standard radiology report (an example is shown in Fig. 2). To evaluate the variance in GPT-4′s text output and to achieve good coverage of its generative capabilities, two identical follow-up prompts were generated resulting in three simplified reports per original report in total (representative examples per report category are given in the Supplemental Material Appendix S1). The time per report generation was noted and the chat session was restarted before each repetition of the prompt to prevent any potential bias of the generated response via cached tokens.

### 2.3 Recruitment of medical laypersons

Medical laypersons were recruited by direct written invitation, ensuring that only individuals with advanced English proficiency (at least C1 level according to the Common European Framework of Reference for Languages) and without a medical background (medical laypersons) participated in the study. Only data from participants who answered all questions were included (n = 13).

### 2.4 Questionnaire design

Two questionnaires were designed to (I) validate the GPT-4-based simplified reports by cardiovascular radiologists and (II) to evaluate the general understandability of the reports by medical laypersons. Two experienced cardiovascular radiologists (A.I. with 6 years of experience, and D.K. with 5 years of experience) rated the reports on a Likert scale (from 1 “strongly disagree” to 5 “strongly agree”) for understandability, factual correctness, completeness of relevant findings, and serious misinformation with potential harm to the patient. Conspicuous text passages were marked, and missing diagnostic information was noted in a separate text field. Further qualitative analysis based on the experts’ annotations was performed by a third radiologist (B.S.). Therefore, the radiology experts’ annotations were inductively categorized by content (misinterpretation and relativization of medical concepts, inaccurate transformation and missing information, unusual language and unnecessary information, confabulation). Thirteen medical laypersons were asked to read the reports and evaluate their general understandability on a Likert scale (from 1 “strongly disagree” to 5 “strongly agree”) without looking up any terminology. Both cardiovascular radiologists and laypersons could not be blinded to the report versions, because the GPT-4 generated findings had a particular explanatory style as well as direct and indirect quotes from the original report. The complete questionnaires are provided in the Supplemental Material (Appendices S2 and S3).

### 2.5 Readability analysis

The Automated Readability Index (ARI), corresponding to school grades in the United States, was employed to objectively measure the readability of the text. Quantitative text parameters (number of letters, characters, words, sentences, paragraphs, respectively) as well as estimated reading time were assessed using dedicated software (Hemingway Editor, Long LLC, Durham, North Carolina) [27]. To mitigate potential bias in the readability analysis, direct quotes from the standard reports were intentionally removed from the GPT-4 reports prior to the readability analysis (while both patients and radiological experts received the unaltered simplified report for evaluation).

### 2.6 Subgroup analysis with external CMR reports

To account for the potential variability in the structure and language of radiological reports between different clinics, we conducted a subgroup analysis. Following the methodology of our study, we selected 10 external CMR reports from various radiological centers (including different topics such as nonischemic cardiomyopathy, ischemic heart disease, congenital heart disease, structured reports) and modified them accordingly to avoid the disclosure of sensitive personal patient information. In a next step, GPT-4 was used to generate one simplified version for each original report, using the established prompt. In addition to readability analysis using the ARI, the 10 original and the 10 GPT-4 reports were evaluated by five medical laypersons for their understandability and by two radiological experts for completeness, correctness, and lack of potential harm.

### 2.7 Statistical analysis

Prism (version 10.0.2; GraphPad Software Inc., Boston, Massachusetts) and SPSS Statistics (version 27; SPSS Inc., IBM, Armonk, New York) were used for statistical analysis. Normality assumptions were assessed by visual inspection of the data distribution supplemented by the Shapiro-Wilk test. Continuous variables are presented as mean ± standard deviation and discrete variables as median with interquartile range (IQR). Group comparisons of the readability score and the understandability of the texts were done using linear mixed-effects models. The report ID and participants’ ID were included as random intercepts to account for non-dependencies. To account for the ordinal nature of the Likert Scale outcome, the robustness of the results of the linear mixed-effects model was assessed by Fishers exact tests, separately applied to each of the reports using only one of the repetitions of the GPT-4 reports. The resulting 60 p-values (one for each report-repetition vs expert comparison) were adjusted for multiple testing by the Bonferroni-Holm method. As an additional sensitivity analyses, the understandability outcome was dichotomized (<3 vs ≥3) and a logistic mixed-effect model was fitted to the data. Results are presented by median (IQR) and the p-values of the group estimate of the linear mixed model. Intraclass correlation coefficient (ICC; model: two-way mixed-effects; type: mean of k raters; definition: consistency) were calculated to estimate interrater agreement between the ratings of both radiologists and the test-retest reliability of the three GPT-generated reports. Interpretation of ICCs was defined as follows: almost perfect: >0.80, substantial: 0.61–0.80, moderate: 0.41–0.60, fair: 0.21–0.40, poor: 0.00–0.20 [28]. A linear mixed-effects model was used to compare the data between the four topic groups of the generated reports as described above. To investigate possible associations between the understandability of radiological reports and the age, educational level, and gender of the layperson cohort, the Kendall rank correlation coefficient was employed. The level of statistical significance was set to p < 0.05.

## 3 Results

### 3.1 General report parameters

GPT-4 required an average of 52 s ± 13 (minimum: 8 s; maximum 78 s) to generate simplified reports. The generated reports had more words (361 ± 67 vs 216 ± 89, p < 0.001) and thereby exhibited longer calculated reading times (86 s ± 16 vs 51 s ± 21; p < 0.001) compared to original reports. Further quantitative report parameters are summarized in Table 1.

**Table 1:** General report parameters based on the Automated Readability Index for original and simplified reports.

Variable	All reports (n = 80)	Original reports (n = 20)	GPT-4 reports (n = 60)	p-value
Readability grade	6.2 ± 2.6	10.1 ± 1.9	5.0 ± 1.3	<0.001
Calculated reading time (s)	77 ± 23	51 ± 21	86 ± 16	<0.001
Characters	1924 ± 516	1495 ± 585	2066 ± 404	<0.001
Words	325 ± 96	216 ± 89	361 ± 67	<0.001
Sentences	33 ± 11	24 ± 11	36 ± 9	<0.001
Paragraphs	20 ± 10	13 ± 13	22 ± 8	0.011

*GPT-4: generative pre-rained transformer 4.* Data are means ± standard deviation.

### 3.2 Automated Readability Index

Compared to the standard reports, the ARI score of the GPT-generated versions was significantly lower (10 [9–12] vs 5 [4–6]; p < 0.001). Eighteen out of 20 (90%) standard CMR reports and 2 out of 60 (3%) GPT reports had an ARI score corresponding to the 8th grade level or higher.

### 3.3 Understandability

**Fig. 3:**
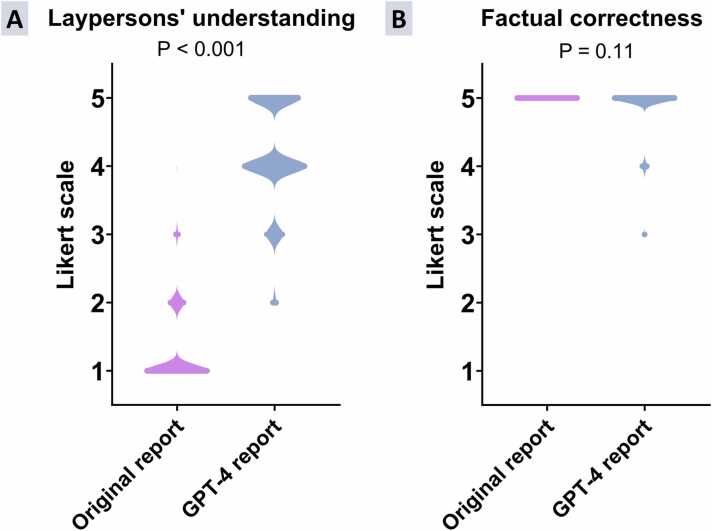
Truncated violin plots illustrating differences in (A) understandability and (B) factual correctness between original reports and simplified GPT-4 (generative pre-trained transformer 4) versions. Data include all layperson (original report ratings: n = 260 [20 reports × 13 laypersons]; GPT-4 report ratings: n = 780 [20 reports × 3 GPT-4 repetitions × 13 laypersons]) and expert (original report ratings: n = 40 [20 reports × 2 experts]; GPT-4 report ratings: n = 120 [20 reports × 3 GPT-4 repetitions × 2 experts]) ratings. Likert scale asking the participant to rate the following statement: (A) “I fully understand the report” and (B) “The modified radiological report is factually correct” (1 = strongly disagree, 5 = strongly agree).

**Fig. 4:**
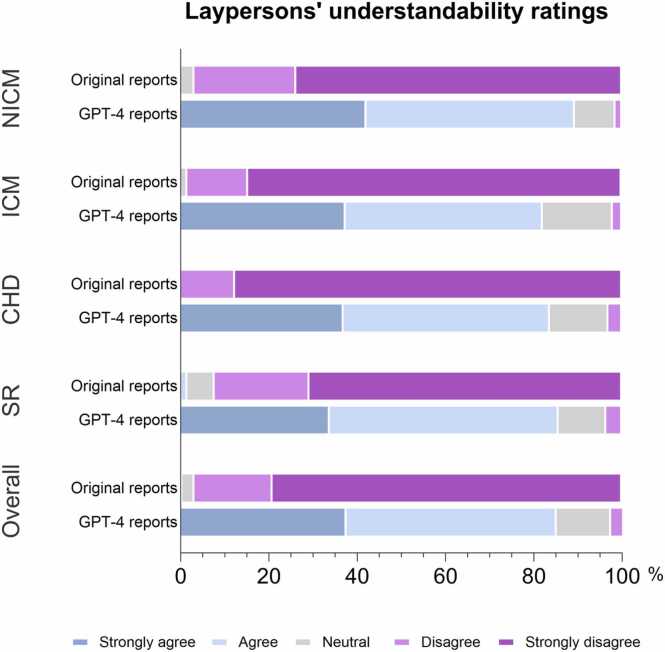
Frequency of the laypersons’ understandability ratings for original and GPT-4 (generative pre-trained transformer 4) report versions according to the different thematic categories. NICM: nonischemic cardiomyopathy, ICM: ischemic cardiomyopathy, CHD: congenital heart disease, SR: structured reports.

Thirteen medical laypersons (7 females and 6 males; 5/13 (38.5%) <30 years old) participated in the study (detailed participant information available in Table S1). Compared to the standard radiology reports, medical laypersons reported a significantly better understanding of the GPT-4-generated reports (1 [1] vs 4 [4,5]; p < 0.001); findings are summarized in Fig. 3. No significant differences were found between the four categories of CMR reports (p = 0.28; Fig. S1). The frequency of the understandability ratings according to the different report categories is provided in Fig. 4. Cardiovascular radiologists reported a high understanding of both the standard and the GPT-generated reports (5 [5] vs 5 [5]). Detailed results for the understandability ratings are provided in Table 2.

**Table 2:** Results of the medical laypersons (n = 13) and cardiovascular radiologists (n = 2) questionnaires for different parameters according to standard cardiovascular magnetic resonance reports (n = 20) and GPT-4 generated reports (n = 60).

Report	Group	Parameter	Median	Q1	Q3	IQR	Mean	SD	Min	Max
Standard reports	Layperson	Understandability	1	1	1	0	1.24	0.51	1	4
	Radiologist	Understandability	5	5	5	0	5.00	0.00	5	5
		Factual correctness	5	5	5	0	5.00	0.00	5	5
GPT-4 reports	Layperson	Understandability	4	4	5	1	4.20	0.75	2	5
	Radiologist	Understandability	5	5	5	0	5.00	0.00	5	5
		Factual correctness	5	4	5	0	4.91	0.32	3	5
		Completeness	5	5	5	0	4.83	0.42	3	5
		No potential harmful conclusion	5	5	5	0	4.86	0.44	3	5

Likert scale: 1 - strongly disagree, 5 - strongly agree; GPT-4, generative pretrained transformer 4

### 3.4 Factual correctness, completeness, and potential harm

**Fig. 5:**
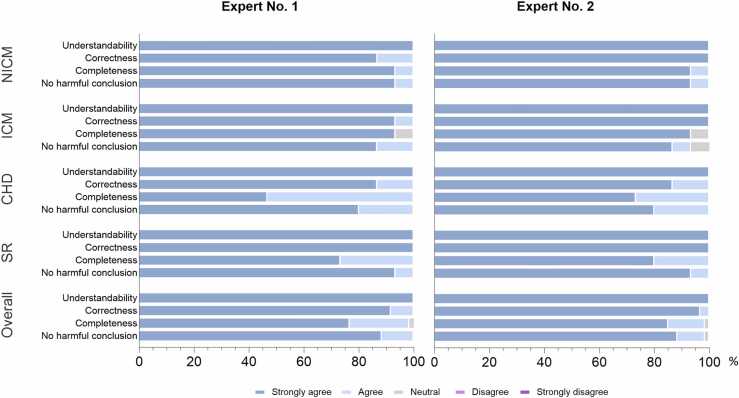
Radiologists’ evaluations according to the report category. NICM: nonischemic cardiomyopathy, ICM: ischemic cardiomyopathy, CHD: congenital heart disease, SR: structured reports.

Cardiovascular radiologists widely agreed that the GPT versions were factually correct (4.91 ± 0.32). There was no significant difference between the factual correctness of the original reports and the GPT-4 versions (5 [5] vs 5 [4,5]; p = 0.11). In about 94% of ratings (113/120), the cardiovascular radiologists “strongly agreed” that the GPT-4 report version was factually correct, and there were no reports where the answers “disagree” or “strongly disagree” were chosen. In about 81% (97/120) of ratings, radiologists reported “strong agreement” for completeness of relevant findings. Summary results for factual correctness, completeness, and potential harm rated by both radiologists are presented in Table 2. Detailed evaluations of the individual experts according to the report category can be found in Fig. 5. Inter-rater reliability of both radiological experts was almost perfect concerning the assessment of potential harm (ICC: 0.93, p < 0.001). Moderate to substantial inter-rater agreement was found for the assessment of completeness (ICC: 0.76, p < 0.001) and factual correctness (ICC: 0.55, p < 0.001).

### 3.5 Qualitative analysis

In total, the radiology experts marked 25 out of 2252 sentences (0.01%) for factual incorrectness or lack of relevant medical information (Expert 1: 9 sentences; Expert 2: 15 sentences), and 9 out of 2252 sentences (0.004%) for potentially harmful conclusions to the patient (Expert 1: 5 sentences; Expert 2: 4 sentences). For the qualitative analysis, the radiology experts’ annotations were inductively categorized by content. The issues specified in the following were identified in the GPT-4 reports.

#### 3.5.1 Misinterpretation and relativization of medical concepts

The most noted discrepancy between the original and the generated reports was the misinterpretation of medical terms (21/34 annotations [61.8%]). This manifested mostly in the form of downplaying and relativizing findings of the original report, exemplified in a report where left ventricular hypertrophy was relativized as “IVST up to 19 mm: [.] it's a bit thick.” In a few cases, experts assessed these downplays as potentially dangerous for the patient, e.g., findings of subacute myocardial ischemia were first correctly explained in the impression as “The findings suggest there's scar tissue […] the condition is likely due to other causes, referred to as myocardial infarction with non-obstructed coronary arteries but at the end relativized as “The heart's left side looks mostly normal, but there are slight abnormalities in its function.”

#### 3.5.2 Inaccurate transformation and missing information

In some instances, the simplification of content led to passages with imprecise language. The finding “New onset of active pericarditis with subsiding pericardial effusion, most likely consistent with Dressler syndrome” was simplified to “The changes in the protective sac around the heart are likely due to a condition called Dressler syndrome, [.],” without mentioning the existing pericardial inflammation (pericarditis) and pericardial effusion.

#### 3.5.3 Unusual language and unnecessary information

In a few reports, inappropriate language was observed. In a report the finding “No regional wall motion abnormalities” was explained as “[.] no parts are being lazy [.].” Experts found some information to be partially superfluous, e.g., a unit conversion was added “[.] blood clot [.], measuring about 40 by 16 mm (for reference, 25.4 mm is 1 in.).”

#### 3.5.4 Confabulation

In the simplified reports, no instances of confabulations were observed by the cardiovascular radiologists.

### 3.6 Reliability of the GPT-4 output using the same prompt

Test-retest agreement showed a substantial correlation between the three generated GPT-4 reports generated from the same original report in terms of layperson understandability (ICC: 0.62, p < 0.001) and ARI score (ICC: 0.59, p = 0.009). Moderate correlations were also found for factual correctness of the simplified reports assessed by radiological experts (ICC: 0.53, p = 0.003).

### 3.7 Subgroup analysis with external CMR reports

Compared to the original reports that were created according to the reporting structure of external centers, the GPT-4 reports were significantly better understandable for the study participants (1 [1, 2] vs. 5 [4, 5]; p < 0.001) (Table S1). They had a significantly lower ARI score (10.2 ± 2.0 vs 7.8 ± 0.9; p = 0.014) (Table S2) while predominantly maintaining the factual correctness (4.85 ± 0.37), completeness (4.90 ± 0.31), and lack of potential harm (5 ± 0) assessed by experts (Table S1).

## 4 Discussion

The main results of this study are that GPT-4 can effectively simplify and explain CMR reports to medical laypersons while largely maintaining factual correctness and completeness. GPT-4 reports significantly improved the subjective understanding for non-medical professionals compared to the standard CMR reports, supported by achieving a lower ARI-based readability grade level. High expert ratings for correctness, completeness, and lack of potential harm were achieved for GPT-4 reports.

Adequate comprehension of medical reports is essential for successful patient-centered health care [21]. Numerous studies, particularly in the field of radiology, indicate that patients struggle to understand conventional reports, which are primarily intended for referring physicians [14], [15], [16], [18]. Insufficient understandability of one's own health condition has been empirically linked to reduced patient compliance, subsequently leading to diminished treatment efficacy [29], [30]. Recent scientific attention has increasingly focused on assessing the effectiveness of various LLMs, including GPT in simplifying medical reports [23], [24]. Previous feasibility studies examining GPT-based text simplification for radiological reports have focused on simplifying general radiological reports across different modalities [25], assessing consistency in repeated report transformations using the same prompt [24], or exploring different prompts for report generation [24], [26]. In recent comparable studies investigating the generative capabilities of GPT in simplifying medical information, a higher patient understandability of GPT-generated reports was observed [31], [32]. However, these studies did not explore whether GPT-4 can facilitate simplification of complex subspecialized reports.

CMR reports represent a unique complexity due to their specialized technical and anatomical language, diverse tissue characterization parameters, and numerous functional and quantitative measures [19], [20], [33]. CMR reports of congenital heart disease are considered particularly complex [34]. Overall, CMR findings may be difficult to understand not only for medical laypersons but even for radiologists without a cardiovascular background or physicians from other specialties. Also, studies have revealed that comprehension problems arising from complex reports also exist between radiologists and clinicians, hindering effective communication [35].

Our findings are widely consistent with previous studies on text simplifications using GPT models [24], [26], while in addition directly assessing the understandability of simplified reports by medical laypersons. Prior studies used a readability index as a measure to quantify understandability [16], [25], [35]. To ensure patients can comprehend information about their health, the National Institutes of Health and the American Medical Association recommend a readability level at the sixth-grade level for medical information [22]. The average readability of CMR reports based on the ARI was substantially lowered from the 10th to the 5th grade level by using GPT-4. However, good readability does not necessarily equate to good understandability. Particularly, the original reports of the categories congenital heart disease and structured reporting demonstrated comparatively good readability but were rated similarly poorly for understandability by medical laypersons. As an example, the two simplified reports with a readability grade of 8 were simplified versions of the same original report from the category of structured reports. Despite the relatively high ARI score, the mean understandability of laypersons for these reports was good (mean 3.8 out of 5 and 4 out of 5, respectively) and did generally not differ from the evaluations of other reports generated by GPT-4. This discrepancy can be explained by the calculation of the ARI that relies on the factors characters per word (indicating word difficulty) and words per sentence (indicating sentence difficulty).

A previous study comparing the effectiveness of GPT-3 and GPT-4 in simplifying radiological reports found substantial variability among report outputs when the same prompt was used, particularly when using GPT 3 [24], [26]. While specific details about the GPT-4 model architecture and training process are not available in OpenAI's current technical report, GPT-4 claims to offer improved accuracy, reduced confabulation, and better understanding of nuanced instructions compared to GPT 3.5 [10], [11]. We did not observe any significant differences in terms of understandability, readability, or factual correctness and completeness between the three versions generated with the same GPT prompt. The extent to which these results can be further improved through prompt design should be explored in subsequent studies.

In this study, only the initial output after entering a prompt was used for further evaluation. The evaluation of the chatbot feature of GPT-4 was not covered in this study. In a real-life scenario, a medical layperson who does not understand a particular text passage or term in the simplified GPT report might ask for a more detailed explanation of that specific point. Therefore, the understandability when using the chatbot function could be even higher than measured in our study. In a previous study, the chatbot function of GPT was examined through interview-based evaluations [36] and GPT's capabilities were considered promising for supporting and enhancing the learning experiences of nuclear medicine students as well as providing valuable support in clinical practice. The chatbot function also has great potential for improving the understandability of complex medical reports and should be explored in future studies.

Due to its accessibility and availability, the use of GPT to explain medical reports is already a real-life scenario today. Although health care providers are generally restricted in using non-local online models due to privacy protections, patients are free to enter their own medical data into commercial models, such as GPT to generate simplified reports. However, it is crucial to emphasize that simplified reports cannot serve as a full substitute for a physician consultation. Previous studies have already revealed that GPT might occasionally add invented information (confabulations) or omit crucial medical information in the simplified report [26]. In our study, a few cases of omission were observed, whereby the generated reports produced downplayed medical findings, which may have been considered a potential risk by radiologists. Such errors suggest that uncontrolled use of GPT or other available LLMs may carry potential risks and that automated simplified results need to be reviewed by professionals if used in clinical practice [3], [37]. However, when GPT-4 is used as a complementary tool by patients who are aware of potential errors, it can generally provide patients with a better understanding of their own health status and has the potential to open up new avenues for patient-physician communication, thereby improving patient compliance.

## 5 Limitations

Our study has some limitations. This feasibility study has an exploratory design and is hypothesis-generating. The sample size of medical laypersons is relatively small, which cannot represent the full range of patients. Both laypersons and experts could not be blinded because the GPT-4 generated reports included an explanatory style as well as direct and indirect quotes from the original report. During the questionnaire completion process, participants may have experienced a learning effect or questionnaire fatigue as they read through the simplified reports, potentially influencing their subsequent assessment of understandability. Consequently, the already poorly rated understandability of the original reports may have been even underestimated. In addition, the possibilities of GPT to specifically adapt to the respective layperson were not examined in our study (e.g., specific prompts such as “explain the findings to a 5-year-old” would be possible). However, the aim of our pilot study was to investigate which results GPT provides when just general prompts are used.

### 5.1 Conclusion

This study demonstrates the potential of GPT-4 in simplifying CMR reports by transforming them into understandable and easily accessible information for medical laypersons, while overwhelmingly maintaining factual correctness and completeness. These results highlight current opportunities in the application of LLMs in radiology and may contribute to improved patient communication and a more patient-centered care in the future.

## Funding

A.I. was funded by the BONFOR Research Commission of the Medical Faculty Bonn (BONFOR-Forschungskommission der Medizinischen Fakultät Bonn) and by the 10.13039/501100001659German Research Foundation (Deutsche Forschungsgemeinschaft, DFG) under Germany’s Excellence Strategy (EXC2151-390873048).

## Author contributions

**Daniel L. Kuetting:** Writing – review and editing. **Ulrike Attenberger:** Writing – review and editing. **Leon M. Bischoff:** Writing – review and editing, Data curation. **Narine Mesropyan:** Writing – review and editing, Data curation. **Anna Odenthal:** Writing – review and editing, Data curation. **Leonie Weinhold:** Writing – review and editing, Formal analysis. **Alois M. Sprinkart:** Writing – review and editing, Formal analysis. **Sebastian Nowak:** Writing – review and editing, Formal analysis. **Dmitrij Kravchenko:** Writing – review and editing, Writing – original draft, Visualization, Project administration, Methodology, Investigation, Formal analysis, Data curation, Conceptualization. **Alexander Isaak:** Writing – review and editing, Writing – original draft, Visualization, Supervision, Project administration, Methodology, Investigation, Formal analysis, Data curation, Conceptualization. **Babak Salam:** Writing – review and editing, Writing – original draft, Visualization, Project administration, Methodology, Investigation, Formal analysis, Data curation, Conceptualization. **Julian A. Luetkens:** Writing – review and editing.

## Declaration of competing interests

The authors declare the following financial interests/personal relationships which may be considered as potential competing interests: Alexander Isaak reports financial support was provided by BONFOR Research Commission of the Medical Faculty Bonn. The other authors declare that they have no known competing financial interests or personal relationships that could have appeared to influence the work reported in this paper.
