# Supplementary material for: Generative Pre-trained Transformer 4 makes cardiovascular magnetic resonance reports easy to understand
Source: J Cardiovasc Magn Reson. 2024 Mar 7;26(1):101035. doi: 10.1016/j.jocmr.2024.101035 (PMC10981113; doi:10.1016/j.jocmr.2024.101035)
Supplement: Supplementary file 1 — Supplementary material [file mmc1.docx]

**STUDY TITLE: GPT-4 makes cardiovascular magnetic resonance reports easy to understand**

**SUPPLEMENTAL MATERIAL**

**Table S1.** Demographic data of the study population completing the layperson questionnaire regarding comprehensibility of the original and simplified cardiovascular magnetic resonance reports.

|  | Laypersons (n=13) |
| --- | --- |
| Gender |  |
| Male | 6 (46.2%) |
| Female | 7 (53.8%) |
| Age |  |
| 18-30 | 5 (38.5%) |
| 31-50 | 5 (38.5%) |
| 51-70 | 3 (23%) |
| Highest level of education |  |
| High School | 4 (30.7%) |
| College | 2 (15.4%) |
| University | 5 (38.5%) |
| Academic Degree | 2 (15.4%) |
| Language level |  |
| C1 Advanced | 4 (30.7%) |
| C2 Proficiency | 4 (30.7%) |
| Native speaker | 5 (38.4%) |

**Table S2.** Subgroup analysis of ratings by medical laypersons (n=5) and cardiovascular radiologists (n=2) for cardiovascular magnetic resonance reports, which were based on the report structure of external centers (n=10). Likert scale: 1 - strongly disagree, 5 - strongly agree.

| Report | Group | Parameter | Median | Q1 | Q3 | IQR | Mean | SD | Min | Max |
| --- | --- | --- | --- | --- | --- | --- | --- | --- | --- | --- |
| **Standard reports** | *Layperson* | Understandability | 1 | 1 | 2 | 1 | 1.26 | 0.44 | 1 | 2 |
|  | *Radiologist* | Understandability | 5 | 5 | 5 | 0 | 5.00 | 0.00 | 5 | 5 |
|  |  | Factual Correctness | 5 | 5 | 5 | 0 | 5.00 | 0.00 | 5 | 5 |
| **GPT-4 reports** | *Layperson* | Understandability | 5 | 4 | 5 | 1 | 4.66 | 0.47 | 4 | 5 |
|  | *Radiologist* | Understandability | 5 | 5 | 5 | 0 | 5.00 | 0.00 | 5 | 5 |
|  |  | Factual Correctness | 5 | 5 | 5 | 0 | 4.85 | 0.37 | 5 | 5 |
|  |  | Completeness | 5 | 5 | 5 | 0 | 4.90 | 0.31 | 4 | 5 |
|  |  | No potential harmful conclusion | 5 | 5 | 5 | 0 | 5.00 | 0.00 | 5 | 5 |

**Table S3.** Automated Readability Index subgroup analysis based on cardiovascular magnetic resonance reports, which were based on the report structure of external centers. GPT-4, Generative Pre-trained Transformer 4.

| Variable | All reports  (n=20) | Original reports (n=10) | GPT-4  reports (n=10) | P-value |
| --- | --- | --- | --- | --- |
| Readability grade | 9.0 ± 2.0 | 10.2 ± 2.0 | 7.8 ± 0.9 | .014 |
| Reading time (in sec) | 67 ± 35 | 51 ± 42 | 83 ± 15 | .059 |
| Characters | 1732 ± 841 | 1430 ± 1079 | 2034 ± 359 | .074 |
| Words | 278 ± 143 | 212 ± 174 | 343 ± 60 | .059 |
| Sentences | 25 ± 14 | 25 ± 19 | 26 ± 7 | .444 |
| Paragraphs | 17 ± 12 | 18 ± 16 | 17 ± 7 | .953 |

**
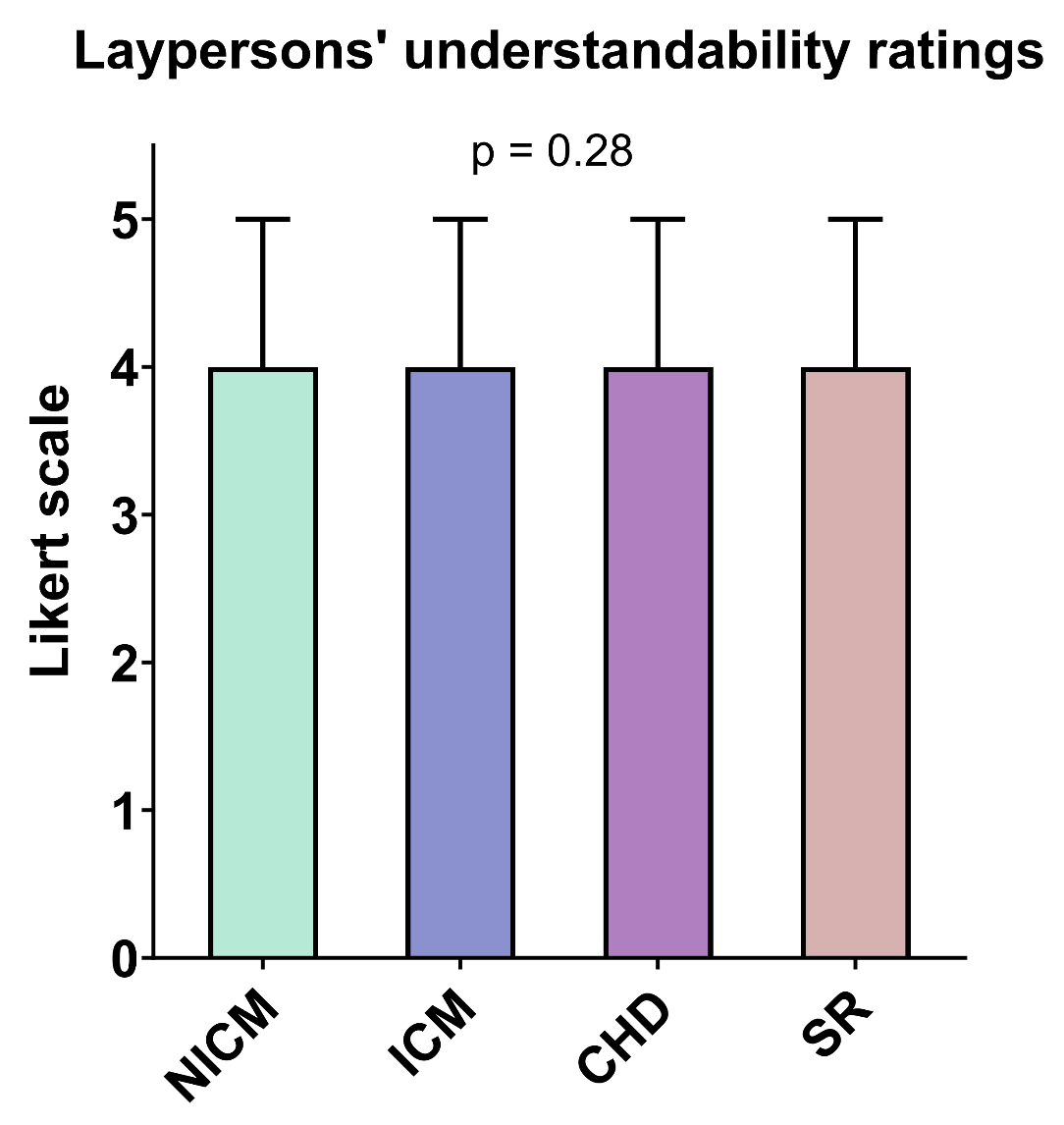
**

**Figure S1.** Box plots (median and interquartile range) represent understandability ratings of medical laypersons stratified in four different thematic categories. P-value is based on linear mixed model with participant and report ID as random intercepts. NICM, nonischemic cardiomyopathy; ICM, ischemic cardiomyopathy; CHD, congenital heart disease; SR, structured reporting.

**Appendix S1.** Exemplary reports of original and simplified versions according to different topics.

***Topic 1: Non-ischemic cardiomyopathy***

**1.0 Original report**

Findings:

No previous cardiac MRI examination available for comparison. Normal left ventricle size (LVEDV: 188 ml; LVEDV/BSA: 103 ml/m²; IVST: 10 mm) with preserved LVEF (57%). No regional wall motion abnormalities. Visual myocardial edema. Increased global T1 (1076 ms; center-specific reference < 1000 ms) and T2 relaxation times (62 ms; center-specific reference < 55.9 ms). Patchy subepicardial enhancement along the entire lateral wall on LGE imaging (series 601, image 5). Competent heart valves on visual assessment. Minimal pleural effusions. Discrete pericardial effusion. Subcutaneous edema of the left axilla.

Impression:

MRI findings consistent with acute myocarditis. No evidence of associated complications.

**1.1 GPT report (Version 1)**

What they looked at:

This is an MRI scan of the heart. There weren't any old MRI scans of the heart to compare with.

What they found:

1. The size of the main pumping chamber of the heart (left ventricle) is normal.

2. The function of the left ventricle is normal and it is pumping well with no areas appearing weak or abnormal.

3. There are signs of swelling in the heart muscle.

4. The MRI measurements show values which are higher than what's typically expected, suggesting potential inflammation or changes in the heart.

5. There's a patchy area of abnormality on the outer layer of the heart muscle, especially along the side of the heart.

6. The heart valves (which control blood flow in the heart) appear to be working fine.

7. There's a small amount of fluid around the lungs and the heart.

8. There's swelling observed in the skin tissue of the left underarm area.

Overall understanding:

The MRI shows findings that suggest an inflammation of the heart muscle called acute myocarditis. Fortunately, there doesn't seem to be any complications related to this condition at this time.

**1.2 GPT report (Version 2)**

Findings:

1. No previous cardiac MRI examination available for comparison: This is the patient's first heart MRI at this facility, so they don't have any older images to compare it to.

2. Normal left ventricle size: The left ventricle (one of the heart's main pumping chambers) is of a normal size. The numbers provided (like LVEDV, LVEDV/BSA, IVST) are just specific measurements that doctors use to determine this.

3. Preserved LVEF (57%): The Left Ventricular Ejection Fraction (LVEF) tells us how well the left ventricle pumps blood with each beat. 57% is within the normal range.

4. No regional wall motion abnormalities: The walls of the heart are moving as they should; no parts are being lazy or not moving correctly.

5. Visual myocardial edema: There's swelling in the heart muscle, which is not usual.

6. Increased global T1 and T2 relaxation times: Without going into the complexities of MRI technology, these numbers suggest some changes in the heart tissue. They're higher than the usual range for this specific medical center.

7. Patchy subepicardial enhancement along the entire lateral wall on LGE imaging: There are areas of abnormality in the outer layer of the heart's wall when they use a particular imaging technique (LGE).

8. Competent heart valves on visual assessment: The heart's valves (which control blood flow) look good.

9. Minimal pleural effusions: A small amount of fluid has built up around the lungs.

10. Discrete pericardial effusion: There's a small amount of fluid around the heart, which shouldn't normally be there.

11. Subcutaneous edema of the left axilla: There's swelling in the tissue under the skin near the left armpit.

Impression:

The MRI results suggest the patient has acute myocarditis, which is inflammation of the heart muscle. Thankfully, the report doesn't show any complications related to this condition.

**1.3 GPT report (Version 3)**

Findings:

- There's no previous heart MRI (a type of imaging) to compare with.

- The size of the left side of the heart (left ventricle) is normal.

- LVEDV, LVEDV/BSA, and IVST are just measurements that show the size and thickness of the heart's left side are within the expected range.

- The heart's pumping ability (LVEF, which stands for "left ventricular ejection fraction") is 57%, which is within the normal range.

- There are no abnormal movements in the walls of the heart.

- There's visible swelling (edema) in the heart muscle.

- T1 and T2 are technical measures from the MRI. They're a bit elevated, suggesting there might be something wrong.

- There's an irregularity (patchy enhancement) on the outer layer of the heart's side wall, which was specifically seen in one of the images taken.

- The heart's valves, which help control blood flow, look good.

- There's a small amount of fluid around the lungs (minimal pleural effusions) and the heart (discrete pericardial effusion).

- There's also some swelling (edema) in the underarm area on the left side (left axilla).

Impression:

- The MRI results show signs consistent with "acute myocarditis," which means there's inflammation (swelling and irritation) of the heart muscle. This can be caused by various things like infections.

- Good news: The MRI doesn't show any complications related to this condition.

***Topic 2: Ischemic heart disease***

**2.0 Original report**

Findings:

No previous examinations available for comparison. Dilated left ventricle (LVEDV: 213 ml, LVEDV/BSA: 116 ml/m², IVST: 14 mm) with moderately reduced function (LVEF: 38 %). Visually mild mitral valve regurgitation. Normal global T1 and T2 relaxation times (T1: 978 ms, reference < 1000 ms; T2: 52 ms, reference <55.9 ms). Predominantly transmural enhancement at the basal anteroseptal/anterior/anterolateral/inferolateral heart segments with a 50-75% transmural scar at the midventricular anterior/anterolateral/inferolateral and apical anterior/lateral heart segments including the apex. Associated thinning of the left ventricular wall with hypo- to akinesia. No evidence of a thrombus. No evidence of myocardial hypoperfusion under adenosine stress. No pleural or pericardial effusions.

Impression:

Ischemic cardiomyopathy with mild dilatation and moderately reduced systolic function of the left ventricle. 50-75% transmural up to complete transmural ischemic scarring of the left ventricle (LAD and RCX territory). No evidence of stress-induced myocardial ischemia beyond the ischemic scar.

**2.1 GPT report (Version 1)**

Findings:

1. Dilated left ventricle: The left chamber of the heart (left ventricle) is enlarged. The specific measurements provided (LVEDV, LVEDV/BSA, and IVST) are technical details indicating the size and thickness of the heart's walls.

2. Moderately reduced function: The heart's ability to pump blood is somewhat reduced. The LVEF of 38% is a measure of this, and a normal value is usually 55% or higher.

3. Mild mitral valve regurgitation: A little bit of blood is leaking backward through the heart's mitral valve each time it closes.

4. Normal global T1 and T2 relaxation times: These are MRI values that give information about the heart tissue's health. In this case, they're within normal limits.

5. Transmural enhancement & associated scarring: Parts of the heart muscle show evidence of damage that affects most or all of the thickness of the wall. This is seen in various areas of the heart, and some areas have scars covering 50-75% of the wall.

6. Thinning of the left ventricular wall with hypo- to akinesia: The heart wall has become thin in some areas, and these areas don't move as well (or at all) when the heart beats.

7. No evidence of a thrombus: No blood clots are seen in the heart.

8. No evidence of myocardial hypoperfusion under adenosine stress: When the heart was put under stress using a drug called adenosine, there was no evidence that parts of the heart muscle were not getting enough blood supply.

9. No pleural or pericardial effusions: There is no abnormal fluid buildup around the lungs (pleural) or the heart (pericardial).

Impression:

This is a summary of the findings. The left ventricle of the heart is enlarged, and its pumping ability is moderately reduced. There are areas of damage and scarring in the heart muscle, specifically in regions supplied by the LAD (Left Anterior Descending) and RCX (Right Circumflex) arteries. However, there's no new evidence of reduced blood flow to the heart muscle beyond the areas that are already scarred.

In simpler terms, there is evidence of previous damage to the heart muscle, likely due to reduced blood flow in the past, and this has affected the heart's ability to pump blood efficiently. However, there are no new areas of concern or current issues with blood supply to the heart muscle.

**2.2 GPT report (Version 2)**

Findings:

1. No previous examinations available for comparison: There's no older test to compare with the current one, so they're just looking at what they see now.

2. Dilated left ventricle: The main pumping chamber of the heart (on the left side) is enlarged.

3. Moderately reduced function: This same pumping chamber isn't working as well as it should. It's only working at 38% of its full potential (that's what the 38% means).

4. Mild mitral valve regurgitation: The mitral valve is a door-like structure in the heart that makes sure blood flows in the right direction. In this case, it's letting some blood leak backward, but it's a mild case.

5. Normal global T1 and T2 relaxation times: These are measures of heart muscle health using an MRI. Both values are within the normal range.

6. Predominantly transmural enhancement ... including the apex: This is a complex way of saying that there's a significant area of the heart muscle that shows signs of past damage. This damage covers a large part of the muscle's thickness in various regions.

7. Associated thinning of the left ventricular wall with hypo- to akinesia: Parts of the heart muscle wall have become thin, and these parts are either moving very little or not at all.

8. No evidence of a thrombus: There's no sign of a blood clot in the heart.

9. No evidence of myocardial hypoperfusion under adenosine stress: When the heart was put under stress using a drug called adenosine, there were no signs of reduced blood flow beyond the already identified damaged areas.

10. No pleural or pericardial effusions: There's no fluid accumulation around the lungs or heart, which is a good sign.

Impression:

The main takeaway from this report is that the patient has a condition called "Ischemic cardiomyopathy." This means that, at some point, parts of the heart muscle didn't get enough blood, leading to muscle damage. The left ventricle, one of the main pumping chambers of the heart, is slightly enlarged and isn't pumping as effectively as it should be. A significant portion of the heart muscle shows damage, especially in areas supplied by two major heart arteries (LAD and RCX). Thankfully, under stress, there's no new area of reduced blood flow, and there's no sign of a clot or fluid accumulation around the heart or lungs.

**2.3 GPT report (Version 3)**

Findings:

1. No previous exams available for comparison: They don't have any older reports to see if there have been any changes over time.

2. Dilated left ventricle: The left ventricle, one of the main pumping chambers of the heart, is larger than normal.

3. LVEDV: 213 ml, LVEDV/BSA: 116 ml/m², IVST: 14 mm: These are measurements related to the size and thickness of the heart's walls. They indicate that the heart's left ventricle is enlarged and the wall is thicker than normal.

4. Moderately reduced function (LVEF: 38%): The heart's ability to pump blood is reduced; the left ventricle is only pumping out 38% of the blood it takes in, which is less than ideal.

5. Mild mitral valve regurgitation: The mitral valve in the heart is not closing properly, which allows some blood to flow backward instead of forward.

6. Normal global T1 and T2 relaxation times: These are measures from an MRI test. They are within normal limits, which means there aren't signs of certain types of heart tissue damage.

7. Enhancement and scarring in several heart segments: There are areas of the heart that show damage, specifically scars. The description details where these scars are located.

8. Thinning of the left ventricular wall with hypo- to akinesia: Parts of the heart wall are thin and have reduced movement or no movement at all.

9. No evidence of a thrombus: They didn't find any blood clots in the heart.

10. No evidence of reduced blood flow under stress: When they made the heart work harder using a drug called adenosine, they didn't see any new areas where blood flow was decreased.

11. No pleural or pericardial effusions: There's no excess fluid around the lungs or the heart.

Impression (the overall takeaway):

- Ischemic cardiomyopathy: This means there is heart muscle damage due to reduced blood flow, which can be from blocked arteries or other causes.

- Mild dilatation and moderately reduced function of the left ventricle: The left side of the heart is a bit enlarged and isn't pumping as well as it should.

- Scarring of the left ventricle: There are scars on the heart, which can come from previous heart attacks or reduced blood flow. These scars are in areas that would be supplied by two specific arteries.

- No evidence of stress-induced damage beyond the scar: When they stressed the heart, they didn't find any new areas of damage besides what's already scarred.

In simple terms, this report tells us that there's damage to the heart muscle, likely due to reduced blood flow in the past, resulting in scarring and affecting the heart's ability to pump effectively. However, when they tested for more areas of reduced blood flow, they didn't find any new problems beyond what's already damaged.

***Topic 3: Congenital heart disease***

**3.0 Original report**

Findings:

No previous studies available for comparison. Known Ebstein anomaly and pulmonary valve stenosis with status post recent balloon dilatation of the pulmonary valve. Dilated right ventricle (functional RVEDV/BSA: 107 ml/m², anatomical RVEDV/BSA including arterialized RV: 132 ml/m²) with preserved function (RVEF: 48%). Normal left ventricle size (LVEDV/BSA: 70 ml/m²) with normal function (LVEF: 54%). Calculated total right/left volume index: approximately 2. No regional wall motion abnormalities. The tricuspid valve is displaced apically by approximately 25 mm (from the mitral valve plane) with an anterior opening without visual signs of valve insufficiency (calculated tricuspid valve regurgitant fraction: 4%). Regular return of the systemic veins into the right atrium. Stenosis of the left inferior pulmonary vein between the left ventricular base and the descending aorta. Unremarkable RVOT without evidence of narrowing. The pulmonary valve annulus measures approximately 14 x 11 mm with a retrograde insufficiency jet. No antegrade flow acceleration on visual assessment. Visually competent aortic valve (the ring measures approximately 11 mm). Unremarkable left aortic arch. Normal coronary anatomy.

Phase contrast flow measurements:

Ascending aorta: SV 27 ml, RF 0 %, Vmax 120 cm/s

MPA: SV 25 ml, RF 20%, Vmax 94 cm/s

RPA: SV 17 ml, Vmax 95 cm/s

LPA: SV 7 ml, Vmax 75 cm/s

No shunting (Qp/Qs = approx. 1). Right dominant pulmonary perfusion with RPA:LPA ratio = 2.6.

Impression:

Ebstein's anomaly with dilatation of the functional part of the right ventricle (total right/left volume index: 2) without significant tricuspid valve insufficiency. After balloon dilatation, mild to moderate pulmonary valve regurgitation without residual stenosis. Right dominant pulmonary perfusion. Functional left inferior pulmonary vein stenosis between the left ventricle and descending aorta.

**3.1 GPT report (Version 1)**

Alright, let's break this down into more understandable language.

Findings:

1. No previous studies available for comparison: This is the first time they've done this type of imaging on the patient, so there's no previous image to compare with.

2. Known Ebstein anomaly and pulmonary valve stenosis with status post recent balloon dilatation of the pulmonary valve: The patient has a known heart defect (Ebstein anomaly) and a narrowing of the pulmonary valve (pulmonary valve stenosis). There was a recent procedure where a balloon was used to open up that narrowed valve.

3. Dilated right ventricle: The right side of the heart is larger than typical.

4. Preserved function: Despite being larger, the right side of the heart is working fairly well.

5. Normal left ventricle size and function: The left side of the heart (the main pumping chamber) is of regular size and working as it should.

6. No regional wall motion abnormalities: All parts of the heart muscle seem to be moving as they should.

7. The tricuspid valve is displaced: A valve in the heart (tricuspid valve) is not in its usual place but doesn't seem to be leaking much.

8. Stenosis of the left inferior pulmonary vein: There's a narrowing in a blood vessel that carries oxygenated blood from the lungs to the heart.

9. Unremarkable RVOT without evidence of narrowing: Another part of the heart is looking good without any narrowing.

10. The pulmonary valve annulus measures approximately 14 x 11 mm with a retrograde insufficiency jet: The region around the pulmonary valve has a certain measurement and shows signs of some backward blood flow.

11. Visually competent aortic valve: The main valve that lets blood out from the heart to the body looks good.

12. Normal coronary anatomy: The blood vessels that supply blood to the heart muscle itself are looking normal.

Phase contrast flow measurements: This section talks about the flow of blood in different parts of the heart and its major vessels. Everything seems to be within expected ranges, and there's no abnormal mixing of blood (no shunting).

Impression:

- The patient has Ebstein's anomaly, which is a rare heart defect present at birth.

- The right side of the heart is bigger than the left, which is not typical.

- Even after a procedure to open up a narrowed valve, there's some leakage, but no remaining narrowing.

- There's more blood going to the right lung than the left.

- There's also a narrowing in a vessel that carries oxygen-rich blood from the lungs to the heart.

In simple words, the report provides detailed findings about the structure and function of the heart. There are a few abnormalities noted, some of which have been treated recently, and some observations about how blood is flowing in and out of the heart. The overall impression gives a summary of these key findings.

**3.2 GPT report (Version 2)**

Background Information: The person has a known heart condition called Ebstein anomaly, where a valve in the heart (the tricuspid valve) is not located where it should be. They also had a narrow pulmonary valve (another valve in the heart), and a procedure using a balloon was recently done to widen it.

What the Imaging Found:

1. Right Side of the Heart (Right Ventricle): It's larger than usual. But, it's working okay.

2. Left Side of the Heart (Left Ventricle): It's of a normal size and is working as it should.

3. Tricuspid Valve: It's located a bit further down than it normally should be. However, it seems to be functioning properly without any leak.

4. Pulmonary Vein Issue: There is a narrowing in one of the veins (left inferior pulmonary vein) that carries blood back to the heart from the lungs.

5. Pulmonary Valve: After the procedure, it seems to be leaking a little (that's the regurgitation). But it’s not narrow anymore.

6. Aorta and Other Valves: They all look fine and normal.

7. Flow Measurements: These are just specific measures of how much blood is flowing through some major blood vessels and at what speed. In essence, the measurements indicate that blood is flowing as it should with no unexpected directions (no shunting).

Overall Assessment:

The person has the heart condition Ebstein anomaly, where a part of the heart is bigger than it should be. The valve that was previously widened is no longer narrow but has a mild leak. Also, there is a narrowing in one of the veins that returns blood from the lungs to the heart.

**3.3 GPT report (Version 3)**

Findings:

1. Ebstein anomaly and pulmonary valve stenosis: These are congenital heart conditions, which means they were present at birth. Ebstein's anomaly involves a problem with the tricuspid valve (one of the heart valves), and pulmonary valve stenosis is a narrowing of the pulmonary valve.

2. Balloon dilatation of the pulmonary valve: A procedure was recently done to widen the narrowed pulmonary valve using a balloon.

3. Dilated right ventricle: The right chamber (ventricle) of the heart is larger than usual, but it's still working well.

4. Normal left ventricle size with normal function: The left chamber (ventricle) of the heart is of regular size and working correctly.

5. Tricuspid valve is displaced: The tricuspid valve has moved a bit from its usual position, but there's no sign that it's leaking more than a negligible amount.

6. Stenosis of the left inferior pulmonary vein: There's a narrowing in one of the veins (left inferior pulmonary vein) that brings blood from the lungs back to the heart.

7. Unremarkable RVOT and pulmonary valve: The part of the heart called RVOT (right ventricular outflow tract) looks normal. The opening of the pulmonary valve has some backflow (regurgitation) of blood, but there's no forward blockage (stenosis).

8. Normal coronary anatomy: The blood vessels that supply the heart muscle (coronary arteries) look normal.

Flow measurements:

These are measurements of how blood flows in different parts of the heart and the major blood vessels. They show how much blood moves (SV) and the speed of its flow (Vmax). Everything seems mostly normal here, but there's a bit more blood flow on the right side of the lungs than the left.

Impression:

- There's an issue called Ebstein's anomaly, and the right chamber of the heart is more prominent than usual.

- After the procedure to fix the narrowed pulmonary valve, there's a bit of backflow through the valve, but no remaining narrowing.

- More blood is flowing to the right side of the lungs compared to the left.

- There's a narrowing in a vein (left inferior pulmonary vein) that brings blood back from the lungs to the heart.

In summary, there are some abnormalities related to the heart's structure and blood flow, but many parts of the heart are working normally. Some issues were addressed with a procedure, and it's essential to keep monitoring these conditions.

***Topic 4: Structured reporting***

**4.0 Original report**

Anatomy and function

Left ventricle

Ejection fraction (LV-EF): 61% (reference: 51-79%)

Dimension: normal

LV-EDD: 45 mm (reference: 41-62 mm)

LV-EDV/BSA: 66 ml/m2 (reference: 45-107 ml/m2)

Wall thickness: IVST 9 mm (reference: <11mm)

Wall motion abnormality: "septal bounce sign" on deep inspiration (evident on the real time cine sequences)

Right ventricle

Dimension: normal

Left atrium

Dimension: normal

Right atrium

Dimension: normal

Pericardium: circumferentially thickened (7 mm), consistent with edematous changes

Pericardial effusion: small effusion (max. lateral border width: 9 mm)

Thrombus: no thrombus

Heart valves

Mitral valve: minor central visual insufficiency

Tricuspid valve: low central visual insufficiency

Aortic valve: flow artifacts suspicious for stenosis, no visual insufficiency

Edema imaging/ myocardial mapping

Visual edema: yes, pericardial; suspicion of focal myocardial edema (basal inferolateral)

T2 signal intensity ratio: 1.7 (reference: <1.9)

T1 relaxation time, global: 1028 ms (reference: <1000 ms); elevated

T1 relaxation time, focal midventricular lateral/inferolateral: 1059 ms

T2 relaxation time, global: 58.4 ms (reference: <55.9 ms); elevated

T2 relaxation time, focal midventricular lateral/inferolateral: 67.7 ms

Late Gadolinium Enhancement (LGE)

Hyperenhancement: yes

Vivid circumferential hyperenhancement of the thickened visceral and, especially, the parietal pericardium. "Patchy" subepicardial and midwall enhancement of the basal inferior and inferolateral segments (series 2001, images 18-20).

Other

Bilateral pleural effusions (left side: 7.5 cm with total atelectasis of the lower lobe; right-side: 1.5 cm). Primarily reactive mediastinal lymph node enlargement (up to SAD: 9 mm, e.g., series 201 image 11).

Impression:

Acute pericarditis with mild, diffuse inflammatory myocardial involvement of the LV lateral wall. Signs of increased ventricular interdependence suggestive of a constrictive component (echocardiographic follow-up recommended). Bilateral pleural effusions (left side drainage therapy recommended).

**4.1 GPT report (Version 1)**

Anatomy and function

This section is giving measurements and observations of different parts of the heart.

- Left Ventricle: This is one of the four chambers in the heart. It pumps blood to the entire body.

-Ejection Fraction (LV-EF): This is how much blood the left ventricle pumps out with each heartbeat. 61% is a good value, within the normal range.

-LV-EDD and LV-EDV/BSA: Measurements of the size of the left ventricle. Both are within the normal range.

-Wall thickness: The thickness of the wall of the left ventricle is normal.

-Wall motion abnormality: There's a sign called the "septal bounce" when taking a deep breath, which could be an indication of a specific heart issue.

-Right Ventricle, Left Atrium, and Right Atrium: The sizes of these other heart chambers are normal.

-Pericardium: This is the sac-like structure that surrounds the heart. It's thicker than usual, suggesting it's swollen.

-Pericardial Effusion: There's a small amount of fluid between the heart and the pericardium.

-Thrombus: Good news – no blood clots are found.

-Heart Valves: These are doors in the heart that ensure blood flows in the right direction.

- There are minor issues with the mitral and tricuspid valves.

- There's a suspicion of narrowing in the aortic valve.

- Edema imaging/ myocardial mapping: This looks at swelling and certain characteristics of heart tissues.

- There's swelling in the sac around the heart and potentially a small area of the heart muscle.

- The T1 and T2 measurements suggest there's inflammation in the heart tissue.

-Late Gadolinium Enhancement (LGE): A type of imaging that looks for damaged heart tissue. It found some areas with damage or inflammation.

Other Observations

- Bilateral Pleural Effusions: There's fluid accumulation around both lungs, more on the left side. This could cause breathing problems.

- Reactive Mediastinal Lymph Node Enlargement: Some lymph nodes, which are part of the immune system, near the heart and lungs are slightly bigger than usual. This could be a sign of inflammation or infection.

Conclusion (Impression):

There's inflammation of the sac around the heart and potential involvement of a part of the heart muscle. Some signs suggest the heart's function could be affected by this. There's also fluid around both lungs, especially on the left side. A follow-up test is recommended to check on some of these findings, and draining the fluid from the left lung side is suggested.

In simpler words, there's inflammation around the heart and some damage signs inside the heart. Additionally, there's fluid buildup around both lungs, which may need treatment, especially on the left side.

**4.2 GPT report (Version 2)**

Your heart has different parts, like chambers and valves. This report is essentially taking a closer look at those parts to see if they're working as they should.

Left ventricle (main pumping chamber of the heart):

- Ejection fraction: This is the percentage of blood that's pumped out of the heart every time it contracts. Yours is 61% which is within the normal range.

- Dimension, wall thickness, and volume: All these parameters are within the normal range for your heart.

- Wall motion abnormality: There's a movement (called "septal bounce") that can be seen when you take a deep breath.

Right ventricle (pumps blood to the lungs): Everything seems normal here.

Left and Right atrium (where the heart receives blood): Both are of normal size.

Pericardium: It's a protective sac around your heart. Yours is thicker than usual and appears to be swollen.

Pericardial effusion: There's a small amount of fluid around your heart.

Thrombus: Good news! There are no blood clots in the heart.

Heart valves:

- Mitral and Tricuspid valves: There's a very minor leak when these valves close.

- Aortic valve: There's a hint that this valve might be narrowing (stenosis), but it's not leaking.

Edema imaging/myocardial mapping:

This is like a fancy scan to check if there's swelling in the heart muscles and surrounding areas.

- The results suggest that there's some swelling both around the heart and in a specific area of the heart.

Late Gadolinium Enhancement (LGE):

This technique helps identify damaged areas in the heart.

- Your results show areas that have taken up the contrast agent (gadolinium) indicating potential damage or inflammation, especially around the heart and in specific areas of the heart wall.

Other:

- Bilateral pleural effusions: There's fluid accumulation in the spaces around both lungs, with the left side having more fluid.

- Mediastinal lymph node enlargement: Some of your lymph nodes, which are part of your immune system, are larger than usual near the heart. This might be a reaction to something.

Summary (Impression):

You have an inflammation of the protective sac around the heart, known as acute pericarditis. Additionally, there's some inflammation in a specific area of the heart. There are also signs that the heart might be working a bit harder because of these issues. They suggest checking the heart's function again with an echocardiogram (a type of ultrasound). There's also a recommendation to possibly drain the fluid from the left side of the lungs.

In simpler words: Your heart and its surrounding area are showing signs of inflammation and some fluid accumulation, but many of the heart's measures are still within normal limits. Some follow-up tests and treatments are recommended to ensure everything gets back to normal.

**4.3 GPT report (Version 3)**

Radiology Report Explained for a Layperson:

1. Left Ventricle:

- Ejection fraction (LV-EF): This is a measure of how well the heart pumps blood out to the body. 61% is within the normal range of 51-79%. So, this is good.

- Dimension: The size of the left ventricle is normal.

- Wall thickness: The thickness of the heart's wall is within normal limits.

- Wall motion abnormality: There's a sign called "septal bounce sign" seen when the heart beats and breath is taken in. This can be a sign of certain heart conditions.

2. Right Ventricle: The size is normal.

3. Left Atrium: The size is normal.

4. Right Atrium: The size is normal.

5. Pericardium: This is the sac around the heart. It's thicker than usual, which is consistent with swelling changes. There's also a small amount of fluid around the heart.

6. Heart Valves:

- Mitral valve: There's a minor issue where the valve doesn't close perfectly.

- Tricuspid valve: Similar minor issue as the mitral valve.

- Aortic valve: There's a concern that this valve might have a narrowing (stenosis), but it's not leaking.

7. Edema Imaging/ Myocardial Mapping:

- Visual edema: There's swelling in the sac around the heart and a suspicion of swelling in a specific part of the heart muscle.

- The next few lines with T1 and T2 values show that certain relaxation times of heart tissues are elevated, which might suggest abnormalities or inflammation.

8. Late Gadolinium Enhancement (LGE):

- This method shows how different parts of the heart muscle take up a contrast agent. There are areas of increased uptake in the sac around the heart and in a particular area of the heart muscle.

9. Other:

- Bilateral pleural effusions: There's fluid in the space around both lungs. The left side has a significant amount, causing a part of the lung (lower lobe) to collapse.

- Mediastinal lymph node enlargement: There are slightly enlarged lymph nodes in the chest area, likely due to a reaction.

Impression (Overall Summary):

The heart's protective sac is inflamed, and there's mild inflammation of a part of the heart muscle. There's a hint that the heart might be constricting in its movement, which is why a follow-up with echocardiography (a heart ultrasound) is recommended. There's fluid around both lungs, and it's suggested to drain the fluid from the left side.

**Appendix S2.** Layperson questionnaire

**Study questionnaire - Comprehensibility of radiology reports**

Date: _ _. _ _ ._ _ _ _

Consent

I consent to participate in this anonymized questionnaire and to the publication of my answers.

- Yes
- No

I confirm that I am not a medical / healthcare professional. (required for study participation)

- Yes
- No

What is your English language level? (at least C1 is required for participation in the study)

- C1 Advanced
- C2 Proficiency
- Native speaker

Gender

- Male
- Female
- Non-binary
- Prefer not to say

Age - Where does your age fit in?

| <18 | 18-30 | 31-50 | 51-70 | >70 |
| --- | --- | --- | --- | --- |
|  |  |  |  |  |

Education

- Primary School
- High school
- College
- University
- Academic degree

What this questionnaire is about

The purpose of this study questionnaire is to assess the comprehensibility of radiology / cardiac MRI reports modified by ChatGPT. You will be presented with twenty different cardiac MRI report groups. Each group consists of

(a) one **original radiology report** (No. 1-20)

(b) and three corresponding reports modified by ChatGPT (No. 1.1, 1.2, 1.3 - 2.1, 2.2, 2.3…).

We kindly ask you to read all reports carefully and answer the questions **without looking up any terminology**.

How to answer

For each individual report, please answer the question ("I fully understand the report in its entirety") by ticking the appropriate box.

Please rate **only the understandability** of the findings and not the type of format or the length of the report.

Please **do not compare directly** between the reports, only rate the comprehensibility of the reports individually (one by one as presented to you).

Time

This questionnaire will take you about 120 minutes to complete.

**Report No. 1**

1. I fully understand the original report in its entirety. (1 = strongly disagree, 5 strongly agree)

| Report No. | 1 | 2 | 3 | 4 | 5 |
| --- | --- | --- | --- | --- | --- |
| 1 |  |  |  |  |  |

1. I fully understand the modified report in its entirety. (1 = strongly disagree, 5 strongly agree)

| Report No. | 1 | 2 | 3 | 4 | 5 |
| --- | --- | --- | --- | --- | --- |
| 1.1 |  |  |  |  |  |
| 1.2 |  |  |  |  |  |
| 1.3 |  |  |  |  |  |

**Appendix S3.** Cardiovascular radiologist questionnaire

**Study questionnaire - Comprehensibility of radiological reports**

Date: _ _. _ _ ._ _ _ _

Consent

I hereby consent to participate in this anonymized questionnaire and to the publication of my answers.

- yes
- no

Experience

How many years of experience do you have as a radiologist in cardiovascular imaging?

What this questionnaire is about

The purpose of this questionnaire is to assess the comprehensibility of radiological reports that have been modified using the large language model GPT-4. You will be presented with twenty different report groups. Each group consists of

(a) one **original radiology report** and

(b) three corresponding **modified versions** generated based on the original report.

We kindly ask you to read all reports carefully and answer the questions of this questionnaire **without looking up any terminology**.

How to answer

For each individual report, answer the questions provided after it.

Provide answers to the questions by ticking a number on the Likert scale (1 = strongly disagree, 5 strongly agree), highlighting text passages by using the provided text marker, or by writing your answers in the textbox provided.

If you want to correct your answer, cross out your previous answer clearly, and tick a new number.

**Questions about the original radiological report**

Q1. The original report is completely understandable for me in its entirety. (1 = strongly disagree, 5 strongly agree)

| Report No. | 1 | 2 | 3 | 4 | 5 |
| --- | --- | --- | --- | --- | --- |
| 1 |  |  |  |  |  |

Q2. The original report is factually correct. (1 = strongly disagree, 5 strongly agree)

| Report No. | 1 | 2 | 3 | 4 | 5 |
| --- | --- | --- | --- | --- | --- |
| 1 | ○ |  |  |  |  |

**Questions about the five corresponding modified reports**

Q3. The modified report is completely understandable for me in its entirety. (1 = strongly disagree, 5 strongly agree)

| Report No. | 1 | 2 | 3 | 4 | 5 |
| --- | --- | --- | --- | --- | --- |
| 1.1 |  |  |  |  |  |
| 1.2 |  |  |  |  |  |
| 1.3 |  |  |  |  |  |

Q4. A. The modified radiological report is factually correct. (1 = strongly disagree, 5 strongly agree)

B. Highlight all **incorrect** text passages (if applicable) with a **yellow** **marker pen**.

| Report No. | 1 | 2 | 3 | 4 | 5 |
| --- | --- | --- | --- | --- | --- |
| 1.1 |  |  |  |  |  |
| 1.2 |  |  |  |  |  |
| 1.3 |  |  |  |  |  |

Q5. A. The modified report contains the relevant medical information for the patient. (1 = strongly disagree, 5 strongly agree)

B. List all relevant medical information in the box, which is missing in the modified report (if applicable).

| Report No. | 1 | 2 | 3 | 4 | 5 |
| --- | --- | --- | --- | --- | --- |
| 1.1 |  |  |  |  |  |
| 1.2 |  |  |  |  |  |
| 1.3 |  |  |  |  |  |

1.1

1.2

1.3

Q6. A. The modified report misleads patients to draw wrong conclusions due to serious misinformation. (1 = strongly disagree, 5 strongly agree)

| Report No. | 1 | 2 | 3 | 4 | 5 |
| --- | --- | --- | --- | --- | --- |
| 1.1 |  |  |  |  |  |
| 1.2 |  |  |  |  |  |
| 1.3 |  |  |  |  |  |

B. Highlight all text passages in the modified reports that might lead to potentially harmful conclusions with a **red** **marker pen** (if applicable).
